# The Expression and Clinical Significance of Different Forms of Mer Receptor Tyrosine Kinase in Systemic Lupus Erythematosus

**DOI:** 10.1155/2014/431896

**Published:** 2014-03-20

**Authors:** Huaqun Zhu, Xiaolin Sun, Lei Zhu, Fanlei Hu, Lianjie Shi, Zhanguo Li, Yin Su

**Affiliations:** Department of Rheumatology and Immunology, Peking University People's Hospital, 11 Xizhimen South Street, Beijing 100044, China

## Abstract

*Objective*. To investigate the expression and clinical significance of trans-membrane MerTK (mMer) on circulating CD14+ monocytes/macrophages and soluble MerTK (sMer) levels in plasma in systemic lupus erythematosus (SLE). *Method*. 108 SLE patients and 42 healthy controls were recruited in this study. The expression of mMer on the surfaces of CD14+ monocytes/macrophages was evaluated by flow cytometry (FCM). The sMer levels were measured by ELISA. Real-time quantitative PCR was applied to evaluate the mRNA levels of MerTK and ADAM17. *Results*. Both mMer expression on CD14+ monocytes/macrophages and sMer levels in plasma significantly increased in SLE patients compared to healthy subjects. The frequency of anti-inflammatory MerTK expressing CD14+CD16+ monocytes decreased in SLE. mMer expression was positively correlated with CD163 expression on CD14+ cells. Both the mMer expression on CD14+ monocytes/macrophages and sMer levels in plasma were positively correlated with SLEDAI. Furthermore, more elevated mMer and sMer levels were found in patients with higher SLEDAI, presence of anti-SSA, anti-Sm autoantibodies, and lupus nephritis. *Conclusion*. Both mMer and sMer levels significantly increased in SLE and positively correlated with disease activity and severity. The upregulation of MerTK expression may serve as a biomarker of the disease activity and severity of SLE.

## 1. Introduction

Mer tyrosine kinase (MerTK) is an integral membrane protein that is preferentially expressed in hematopoietic lineages such as monocytes/macrophages, dendritic cells (DCs), and natural killer (NK) cells [1, 2], which is one of the three members of TAM (Tyro3, Axl, Mer) family receptor tyrosine kinases [3, 4]. The proteolytic cleavage of the extracellular domain of transmembrane MerTK (mMer) by A Disintegrin And Metalloproteinases domain 17 (ADAM17) leads to the production of the soluble form of MerTK protein (sMer), which is released into circulation and inhibits efferocytosis and platelet aggregation [5–7]. MerTK is a key molecular for tolerance maintenance of central and peripheral autoimmune responses through multiple mechanisms including recognition and clearance of apoptotic cells (ACs)-derived autoantigens [8–10], downregulation of TLR-induced production of inflammatory cytokines [11, 12], prevention of abnormal activation of antigen presenting cells [13], and expansion of autoreactive B and T cells [14, 15]. Its functional impairment leads to defective AC clearance and promotes autoimmunity, resulting in lupus-like autoimmune disease [12, 16–19].

Systemic Lupus Erythematosus (SLE) is an autoimmune disease with multiorgan damage characterized by defective phagocytosis of ACs, release of inflammatory cytokines, and aberrant activation of autoreactive T and B cells, with subsequent production of pathogenic autoantibodies against cell nuclear components and resultant end-organ injury [20]. Impaired AC clearance by monocytes/macrophages was critical in SLE pathogenesis, which leads to autoantigen accumulation, presentation, and subsequent autoantibody production and inflammatory response [21, 22]. Cohen et al. demonstrated that Mer-deficient mice showed impaired apoptotic cell clearance and progressive lupus-like autoimmunity [23, 24]. Soluble form of MerTK and its ligand Protein S have been shown to be positively correlated with disease activity in patients with SLE [25, 26]. MerTK is also expressed by CD14+ circulating monocytes/macrophages which are involved in the pathogenesis of SLE [27–29]. These studies suggest that MerTK might contribute to the pathogenesis of SLE by regulating autoimmune response. However, the expression pattern abnormality of mMer and sMer in SLE as well as their clinical relevance in SLE has not been fully elucidated. In order to further investigate the contribution of MerTK in SLE pathogenesis, it is necessary to reveal the expression patterns and clinical significance of MerTK in SLE.

In this study, we determined the expression levels of mMer on circulating CD14+ monocytes/macrophages and sMer levels in plasma from patients with SLE and analyzed the clinical significance of both mMer and sMer in SLE. Our study showed that both of the mMer and sMer levels significantly increased in SLE and positively correlated with disease activity and severity. Meanwhile, we investigated the different mRNA expression of MerTK and ADAM17 between SLE patients and healthy controls to further demonstrate possible reason for increased shedding of MerTK in SLE. Furthermore, we determined the frequency and MerTK expression pattern of M2c-like CD14+ (bright) CD16+, CD163+ monocyte/macrophage population in comparison to healthy subjects [28].

## 2. Material and Methods

### 2.1. Patients and Healthy Controls

B samples were obtained from 108 consecutive patients with SLE (94 females, 13 males) enrolled at the ward of the department of rheumatology and immunology, Peking University People's Hospital from July 2012 to February 2013. All patients fulfilled at least 4 of the 2010 American College of Rheumatology (ACR) revised criteria for SLE [30]. 42 healthy controls were collected from healthy staffs in our hospital. Flow analysis was performed from 42 of the 108 patients and 25 of the 42 healthy controls. Our study was approved by the ethics committee of Peking University People's Hospital. All patients obtained informed consent to donate their blood samples and clinical information for research, and written consent was given from all the patients.

### 2.2. Clinical Data Analysis

General and Laboratory data from the medical records of these patients include age, gender, disease duration, clinical symptoms, blood cell counts (Leucocyte: WBC; Hb: Haemoglobin; PLT: Thrombocyte), routine chemistry, urinalysis, 24 h proteinuria excretion, lupus associated anto-antibodies (anti-dsDNA Ab: anti-double strand DNA antibody; ANA: Antinuclear Antibody; AnuA: Anti-nucleosome Antibody; SSA: anti-SSA antibody; Sm: anti-Sm antibody; ACL: anticardilipin antibody), immunoglobulins (IgG, IgM, IgA), Complement component 3 (C3), Complement component 4 (C4), and C-reactive protein (CRP). White cell and planet counting less than 4 × 10^9^/L and 100 × 10^9^/L were regarded as leukocytopenia and thrombocytopenia, respectively. Proteinuria was defined as 24 h proteinuria excretion more than or equal to 0.5 g. Anti-dsDNA Ab, ACL, AnuA, C3, C4, IgG, IgM, and IgA were tested by ELISA, with normal ranges of 0–100 IU/mL, 0–12 RU/mL, 0–20 RU/mL, 0.88–2.01 G/mL, 0.16–0.47 G/mL, 6.94–16.18 G/mL, 0.6–2.63 G/mL, and 0.68–3.78 G/mL. ANA, SSA, and Sm were tested by indirect immunofluorescence assay. Positive auto-antibodies of anti-dsDNA Ab, ACL, and AnuA were defined as values more than 100 IU/mL, 12 RU/mL, and 20 RU/mL, respectively. Decreased C3 and C4 were defined as values less than 0.88 G/mL and 0.16 G/mL. CRP was examined by immunonephelometry method. Values more than or equal to 7.9 mg/L were considered positive.

Disease activity was calculated by using the SLE disease activity index (SLEDAI) [31]. Clinical features defined as SLEDAI system were seizure, psychiatric symptoms, encephalosis, visual injury, cranial neuropathy, lupus headache, cerebrovascular insult, vasculitis arthritis, myositis, cylindruria (Hb/RBC cylinder, granular cast), haemoglobinuria (>5RBC/HP), pyuria (>5WBC/HP), and leukocytopenia thrombocytopenia.

### 2.3. Detection of MerTK and ADAM17 Expression by Real-Time Polymerase Chain Reaction (RT-PCR)

Human PBMCs were obtained from the venous blood of 35 SLE patients and 26 healthy controls by Histopaque density gradient centrifugation using human peripheral leukocyte isolation liquid (TBC Science, China). Magnetic separation kit (Miltenyi) was used for human CD14(+) monocytes/macrophages enrichment from PBMCs of another 8 SLE patients and 5 healthy controls by positive selection according to the manufactures' instructions. Purity of CD14(+) cells was >95%. RNA in trizol reagent was extracted from PBMC and CD14(+) cells mentioned above with RNA simple total RNA kit (TIANGEN Corporation, China), and cDNA was synthesized from 1 *μ*g of total RNA by using random oligonucleotides as primers and a RevertAid First Strand cDNA Synthesis Kit (Thermo Science Corporation). Gene expression was assessed for glyceraldehyde-3-phosphate dehydrogenase (GAPDH), MerTK, and ADAM17 using the following primers: GAPDH (sense, 5′-AAGGTGAAGGTCGGAGTCAA-3′, antisense, 5′-AATGAAGGGGTCATTGATGG-3′), MerTK (sense, 5′-GTTTGGAGCTGTGATGGAAGGC-3′, antisense, 5′-CGCTTCAGGAAATCCTCC-3′), and ADAM17 (sense, 5′-CGTTGGGTCTGTCCTGGTTT-3′, antisense, 5′-GATTTCGACGTTACTGGGG-3′). PCR amplification was performed by using SYBR Green assay with the following thermal step: initial denaturation at 94°C for 3 minutes (min), followed by 40 cycles of denaturation at 94°C for 30 seconds (s), annealing at 58°C for 30 s, and extension at 72°C for 30 s. The 7300 Detection System (AB Applied Bio-systems) was used to run quantitative real-time PCR of the samples according to the manufacturer's instructions. Reactions were run in triplicate and generated products were analyzed with the SDS software. mRNA levels were expressed as threshold cycle (CT). For relative quantification, the expression target genes were normalized by expression of GAPDH gene. The data was evaluated as 2^−ΔΔCt^ values. Results were expressed as relative quantity to the control as normalization ratio where all the other samples were compared in terms of their fold difference to the control.

### 2.4. Analysis of Cell-Surface Molecules by FACS

Venous blood samples (4 mL) were obtained from all subjects in purple tubes containing ethylenediamine-tetraacetic acid (EDTA) as the anticoagulant. 100 *μ*L volume of blood was used for each subject. Containing serum components were removed by washing the cells three times in an isotonic phosphate buffer (supplanted with 0.5% bovine serum albumin) by centrifugation at 1600 rmp for 5 min. 50 *μ*L of packed cells was then transferred to 5 mL tubes for staining with monoclonal antibodies. Before staining with antibodies, cells to be used were firstly Fc-blocked by treatment with 5 *μ*L Fc receptor blocking solution (Biolegend, Catalog No.: 422301) per 50 *μ*L of suspension for 15 min at room temperature. The following antibodies were added to the tube: anti-human CD14 (FITC, Biolegend), anti-human CD16 (APC, Biolegend), anti-human CD163 (PerCP-Cy5.5, Biolegend), and anti-human MerTK (PE, R&D Systems). Corresponding negative isotype and fluorochrome-matched controls were used in a separate tube. After following the incubation protocols as recommended by the respective manufacturers, 2 mL 1% flow cytometry solution (Multicience, Cat No. LSB01) for lysing red blood cells was added to the whole cells for 10 min in the dark at room temperature. Then, the cells were washed twice in 4 mL of sample PBS buffer. The cells were resuspended in 400 *μ*L of PBS buffer for final flow cytometric analysis. The stained cells were processed in flow cytometry (BD FACS AriaTM II). A forward scatter-side scatter plot was used to gate lymphocytes, monocytes/macrophages, and granulocytes (Figure 2(a)). The percentage of CD14+CD16+ subtype monocytes/macrophages was determined (Figure 2(a)). Likewise, mMer cell-surface expression on CD14+ and CD14+CD16+ monocytes/macrophages was quantified by means of fluorescence intensity (MFI) (Figure 2(a)). The results were analyzed using FlowJo v7.6.5 (USA).

### 2.5. Assay for Plasma sMer Concentrations

Plasma sMer was determined by a quantitative sandwich enzyme-linked immunosorbent assay (ELISA). Blood examples were collected into EDTA tubes and centrifuged at 1600 rmp for 10 min. The plasma was subpacked and aliquots were stored at −80°C until assayed. The DuoSet development system for sMer (DY6488) was purchased from R&D Corporation (Minneapolis, MN, USA). 96-well plates were coated overnight with MerTK capture antibody. The plates were blocked with reagent diluent containing 1% BSA in phosphate buffered saline (PBS: 137 mM NaCl, 2.7 mM KCl, 8.1 mM Na_2_HPO_4_, 1.5 mM KH_2_PO_4_, PH 7.2–7.4, 0.2 *μ*m filtered.). The plates were washed three times with 0.05% Tween-20 in PBS during each step. An eight-point standard curve was made by 2-fold serial dilution of recombinant proteins and blank controls were reagent diluent alone. No plasma dilution was performed for sMer detection. The antigen was detected by a biotinylated goat anti-human Mer antibody (R&D Corporation, Minneapolis, MN, USA) and streptavidin conjugated to horseradish peroxidase (HRP) (R&D Corporation, Minneapolis, MN, USA). The tetramethylbenzidine (TMB) (Neobioscience, China) was added as the substrate solution and the color reaction was stopped by the addition of 50 *μ*L 2 N sulphuric acid. The absorbance was read at 450 nm with a correction wavelength set at 570 nm using a microplate reader (Bio-RAD, Model no. 550). sMer concentrations were calculated using ELISA calc. regression computer software by creating a standard curve through reducing the blank data to generate a four-parameter logistic (4-PL) curve-fit prepared from 2-fold serial dilutions of recombinant MerTK.

### 2.6. Statistical Analysis

The Statistical Package for Social Science (SPSS) version 16.0 was used to analyze the data. Experimental data were expressed as the mean ± standard deviation and statistical significance between two groups was assessed with the Student's paired *t-test*. Spearman's correlation coefficient was applied to detect correlation between two groups. *P* values less than 0.05 were considered significant.

## 3. Results

### 3.1. Demographic and Clinical Characteristics

Demographic and clinical characteristics of SLE patients and healthy controls are shown in Table 1. 108 SLE patients and 42 healthy controls with matched age and gender were recruited in this study (age: 34.63 ± 12.92 versus 35.5 ± 9.75, *P* = 0.1; gender: *X* ^2^ = 1.234, *P* = 0.124). The SLE patients had mean disease duration of 68.16 months ranging from 1 to 420 and the mean SLEDAI score of these patients was 9.44 ranging from 0 to 48.

### 3.2. mRNA Level of MerTK and ADAM17 in PBMC and CD14+ Monocytes/Macrophages

**Figure 1:**
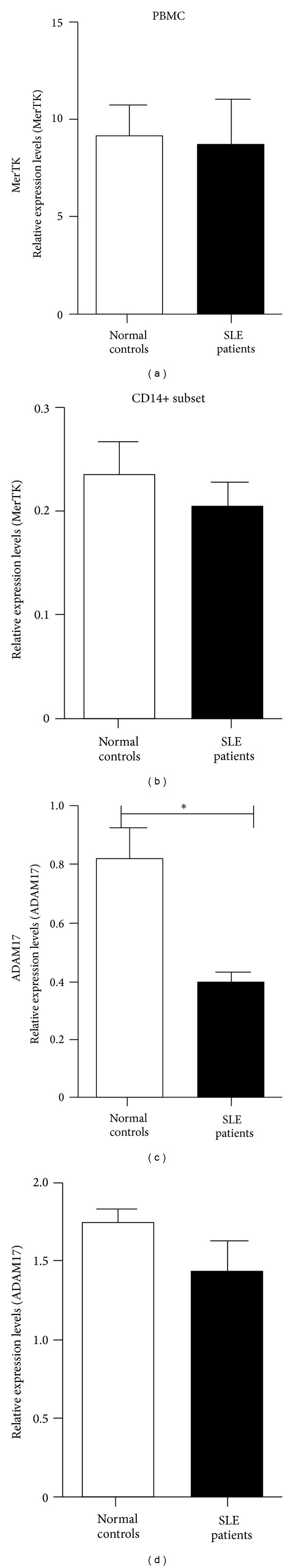
Comparison of gene expressions in PBMC (26 healthy controls and 35 SLE patients for MerTK; 6 healthy controls and 6 SLE patients for ADAM17) and CD14+ monocytes/macrophages. Relative MerTK expression levels in PBMC and CD14+ are shown in (a) and (b), respectively. (c) and (d), respectively, demonstrated the ADAM17 expression in PBMC and CD14+ monocytes/macrophages. Histograms in solid show the relative gene expression in SLE patients compared with expression in healthy controls (histogram in blank). Vertical lines out histograms show standard errors. PBMC: peripheral blood mononuclear cell; MerTK: Mer tyrosine kinase; ADAM17: A Disintegrin And Metalloproteinases domain 17. *P < 0.05.

MerTK and ADAM17 mRNA expression were detected in both SLE patients and healthy controls. As showed in Figures 1(a) and 1(b), there was no significant difference in MerTK mRNA levels in PBMC or CD14+ monocytes/macrophages between patients with SLE and healthy controls (*n* = 35, 8.69 ± 2.28 versus *n* = 26, 9.16 ± 1.6, *P* = 0.876; *n* = 8, 0.20 ± 0.02 versus *n* = 5, 0.23 ± 0.04, *P* = 0.497, resp.). The ADAM17 mRNA level in PBMC was significantly lower in SLE patients than that in healthy controls (*n* = 5, 0.40 ± 0.03 versus *n* = 5, 0.81 ± 0.12, *P* = 0.018). In CD14+ monocytes/macrophages, although the ADAM mRNA levels tended to decrease in SLE patients, there was no significant difference between the patients and the controls (Figures 1(c) and 1(d)). There was a positive correlation between ADAM17 mRNA levels in PBMCs and plasma sMer levels (see  Supplementary Figure 1 in Supplementary Material available online at http://dx.doi.org/10.1155/2014/431896), which implicated that ADAM17 might play a role in promoting Mer shedding and sMer production.

### 3.3. Elevated Expression of MerTK on Circulating CD14+ Monocytes/Macrophages and in Plasma in Patients with SLE

**Figure 2:**
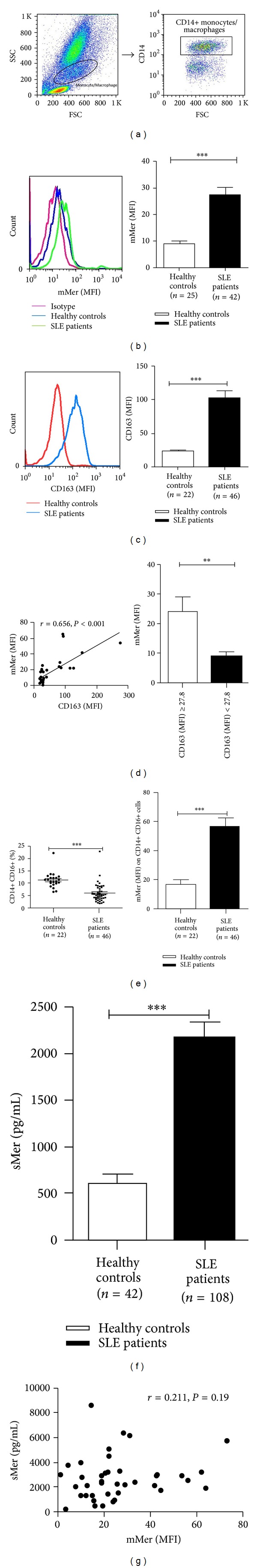
Example of quantification of blood CD14+ monocytes/macrophages and CD14+CD16+ macrophage subset. Membrane MerTK and CD163 expression were measured by flow cytometry as mean fluorescence intensity (MFI). Following monoclonal anti-human Abs were used for detection of FITC-conjugated anti-CD14 Ab, APC-conjugated anti-CD163 Ab, and PE-conjugated MerTK Ab. (a) Cell distribution based on the forward-scatter and side-scatter; the monocytes/macrophages population is identified and gated accordingly. The fraction of monocytes/macrophages positive for CD14 is identified and gated. (b) Histogram showing the MFI of CD14+ monocytes/macrophages positive for MerTK in IgG1 isotype (Purple line), healthy controls (Heavy blue line), and SLE patients (Light green line). mMer expression on CD14+ monocytes/macrophages subset was significantly elevated in patients with SLE compared with healthy controls. (c) CD163 expression on the surface of CD14+ monocytes/macrophages shown by histogram was different between SLE patients (Light blue line) and healthy controls (Red line). Bar showed the more expansion of CD163 expression on CD14+ monocytes/macrophages subset in SLE patients than healthy controls. (d) Correlation between CD163 and mMer expression on CD14+ cells. (e) Characterization of the monocytes/macrophages subsets in PBMC from healthy controls and patients with SLE. The dot plot represented the CD14 and CD16 expression on monocytes/macrophages. Percentages of CD14+CD16+ subset among total monocytes/macrophages were significantly reduced in SLE patients. Circles and squares in solid represented CD14+CD16+ cell frequencies of healthy controls and SLE patients, respectively. CD14+CD16+ monocytes/macrophages subset had elevated mMer expression in patients with SLE in comparison with healthy controls. (f) Comparison about sMer levels in plasma between healthy controls and SLE patients. (g) Correlation between sMer in plasma and mMer on CD14+ cells. The mean ± SD of MFI was shown by bars represented for SLE patients in solid and healthy controls in blank. Horizontal lines above bars showed difference and vertical lines showed standard errors. FSC: forward scatter; SSC: side scatter; mMer: membrane Mer tyrosine kinase; FITC: fluorescein isothiocyanate; APC: allophycocyanin; PE: phycoerythrin; PBMC: peripheral blood mononuclear cell. **P < 0.001, ***P < 0.0001.

The mMer levels on cell surfaces of CD14+ monocytes/macrophages were significantly increased in SLE patients than in healthy controls (*n* = 42, 27.15 ± 2.88 versus *n* = 25, 8.84 ± 1.35, *P* < 0.001) as presented in Figure 2(b).

On CD14+ monocytes/macrophages, we found a significantly elevated CD163 expression in SLE patients than healthy subjects (*n* = 46, 103.66 ± 9.75 versus *n* = 22, 24.83 ± 0.72, *P* < 0.001) (Figure 2(c)). Previous studies reported that mMer expression was mainly restricted to the CD14+CD163+ monocyte subset [28]. Our data showed that CD163 expression on the surface of CD14+ cells was positively correlated to mMer in healthy controls (*r* = 0.656, *P* < 0.001) (Figure 2(d)). We divided healthy subjects into two groups according to the median of CD163 expression on the surface of CD14+ cells in healthy controls. The mMer expression in group with elevated CD163 expression defined as *⩾*27.8 was significantly increased (*P* = 0.008) than that in group with decreased CD163 expression defined as <27.8 in healthy controls (24.11 ± 4.89 versus 8.86 ± 1.69) (Figure 2(d) and Supplemental Figure 2), which is in agreement with the previous study [28].

The CD14+CD16+ subset of human blood monocytes/macrophages, expanding in certain inflammatory conditions, played an anti-inflammatory role in SLE pathogenesis [27]. The percentage of circulating CD14+CD16+ monocyte/macrophage subset was significantly lower in SLE patients compared with healthy controls (6.06 ± 0.53 versus 11.26 ± 0.67, *P* < 0.001). However, significantly elevated mMer expression on this subset in SLE patients was also detected (56.15 ± 6.53 versus 17.18 ± 2.85, *P* < 0.001) (Figure 2(e)).

Our data showed that the elevation of mMer on CD14+ monocyte/macrophages was accompanied by the increased expression of CD163, which defined the CD14+CD163+ subset as the main CD14+ monocyte/macrophage population with elevated mMer expression, and mMer expression in the immune-regulatory CD14+CD16+ subset also increased in SLE.

Besides the elevated expression level of mMer on CD14+ monocyte/macrophages, the plasma sMer level in SLE patients (*n* = 108, 2170.30 ± 160.87 pg/mL) was also significantly higher (*P* < 0.001) than that in healthy controls (*n* = 42, 600.67 ± 115.49 pg/mL) (Figure 2(f)). There was no correlation between the plasma sMer levels and mMer levels on CD14+ monocyte/macrophages (*r* = 0.211, *P* = 0.19) (Figure 2(g)).

### 3.4. Elevated mMer and sMer Levels Were Correlated with Disease Activity and Severe Clinical Manifestations in SLE

**Figure 3:**
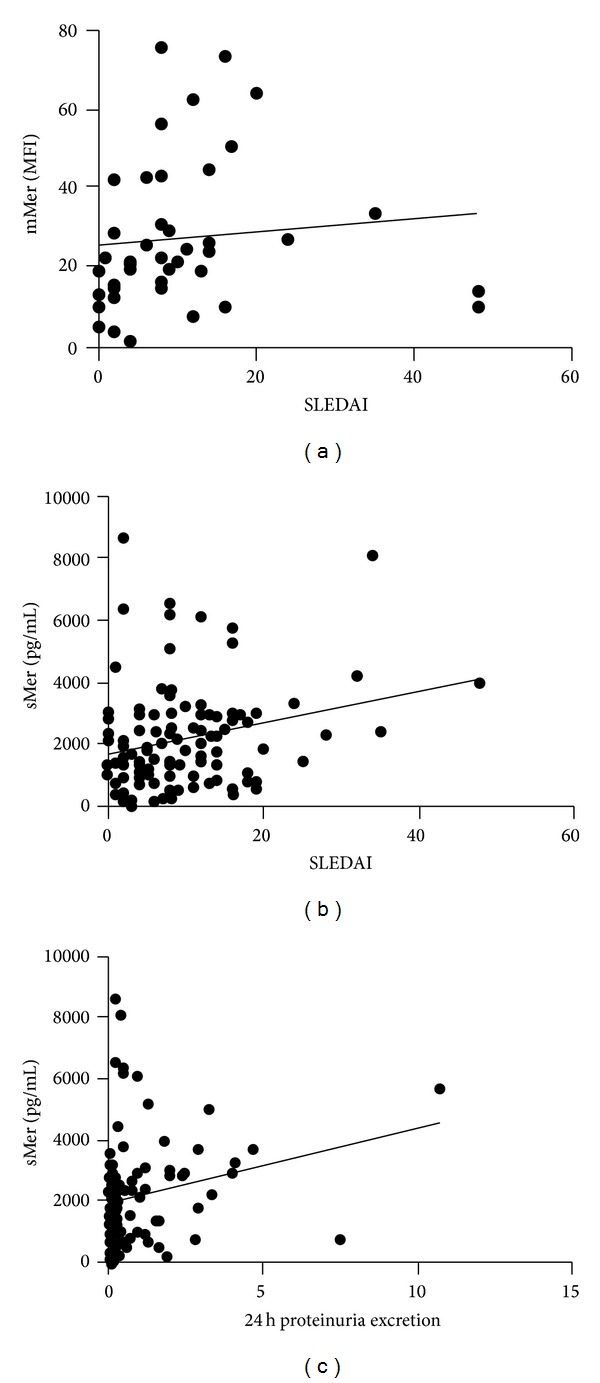
Correlations of mMer and sMer with different clinical parameters such as SLEDAI score and 24 hours proteinuria excretion in patients with SLE. (a) mMer expression on CD14+ monocytes/macrophages was positively correlated to SLEDAI score. (b) sMer in plasma had positive correlation to SLEDAI score, 24 h: 24 hours proteinuria excretion. mMer: membrane Mer tyrosine kinase; sMer: soluble Mer tyrosine kinase; SLEDAI: SLE disease activity index; Spearman's rank correlation test was used to assess correlations.

In patients with SLE, mMer expression on CD14+ monocyte/macrophages was positively correlated to disease activity quantified by SLEDAI score (*r* = 0.343, *P* = 0.026) (Table 2, Figure 3). The plasma sMer concentrations were also positively associated to SLEDAI score (*r* = 0.229, *P* = 0.017) and significant correlation was detected between sMer and 24 hours proteinuria excretion (*r* = 0.320, *P* = 0.002) (Table 2, Figure 3).

**Figure 4:**
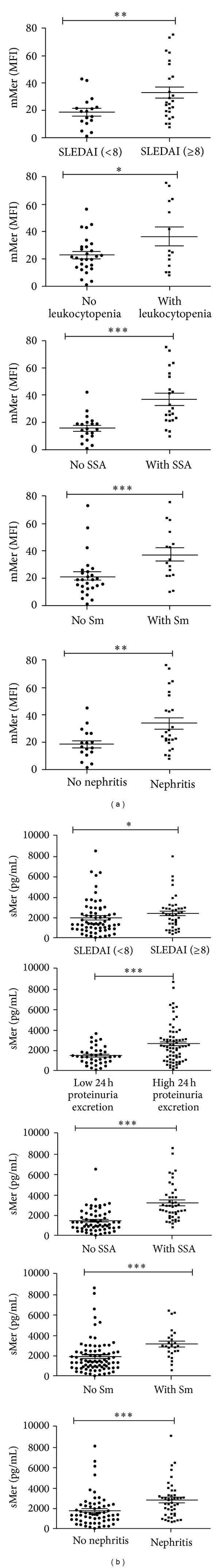
mMer and sMer levels according to the clinical manifestations in SLE. (a) mMer expression on CD14+ monocytes/macrophages in patients with a SLEDAI more than or equal to 8, WBC less than 4 × 10^9^/L, and with SSA, Sm or lupus nephritis. (b) Plasma sMer concentrations in patients with a SLEDAI more than or equal to 8, 24 h proteinuria excretion more than or equal to 0.1 g/d, and with SSA, Sm or lupus nephritis. SLEDAI = SLE disease activity index. WBC: leukocytes; SSA: anti-SSA antibody; Sm: anti-Sm antibody. Circles in solid and in blank represented the mMer or sMer levels in the presence and absence of manifestations in SLE with the studied parameters, respectively. Horizontal lines above dots showed difference. *P < 0.05, **P < 0.01, ***P < 0.001.

Besides the correlation between Mer expression and disease activity, both mMer levels on the surface of CD14+ monocyte/macrophages and circulating sMer levels in plasma were associated with more severe clinical and laboratory manifestations in SLE patients (Figure 4). We grouped patients by SLEDAI or presence of clinical and laboratory features. The patients with SLEDAI not less than 8 showed higher mMer levels on CD14+ monocytes/macrophages (20.19 ± 3.11 versus 4.42 ± 0.64, *P* < 0.001) and sMer levels in plasma (2512.8 ± 221.34 versus 1757.9 ± 222.23, *P* = 0.019). Plasma sMer levels were significantly elevated (*P* < 0.001) in patients with proteinuria (2582.3 ± 225.12 pg/mL) than those without protein excretion (1441.5 ± 139.49 pg/mL). Patients with presence of SSA, Sm, or lupus nephritis showed both higher mMer and sMer levels than those without SSA, Sm, or lupus nephritis (mMer: SSA+ 37.06 ± 4.17 versus SSA− 16.25 ± 2.1, *P* = 0.0001; Sm+ 36.96 ± 4.84 versus Sm− 21.12 ± 3.08, *P* = 0.006; lupus nephritis+ 33.29 ± 4.07 versus lupus nephritis− 18.12 ± 2.72, *P* = 0.004; sMer: SSA+ 3196 ± 267.75 pg/mL versus SSA− 1437.7 ± 139.45 pg/mL, *P* = 0.0001; Sm+ 3123.6 ± 284.85 pg/mL versus Sm−  1868.1 ± 180.00 pg/mL, *P* = 0.001; lupus nephritis+ 2737.1 ± 248.21 pg/mL versus lupus nephritis− 1749.8 ± 196.34 pg/mL, *P* = 0.002) (Table 3, Figure 4).

## 4. Discussion

Deregulation of innate immunity and clearance of apoptotic cells have been implicated in the pathogenesis of SLE [20, 21]. In SLE, cell debris produced by impaired apoptosis may serve as danger signals to break immune tolerance and result in autoimmune inflammation and autoantibody production [22]. As one of the TAM family members, MerTK has been considered to play a vital role in phagocytosis of apoptotic cells and downregulation of inflammatory responses [12, 19]. MerTK knock-out mice are more susceptible to lethal septic shock following lipopolysaccharide (LPS) induction [23]. Mutant mice lacking TAM receptors developed severe lupus-like autoimmune disease induced by impairment of apoptotic cell clearance [12]. Type I IFN signaling triggered by TLR stimulation up-regulated the expression and activation of MerTK, which in turn activated SOCS1/3 signaling and elicited a negative feedback to activated immune response [32, 33]. The downregulation of immune responses by MerTK signaling was an important regulatory mechanism to prevent the rise of autoimmunity.

Membrane MerTK was reported to be shed into soluble forms through ADAM-17 dependent cleavage and circulated in plasma [6]. Although possible defects in MerTK signaling in SLE pathogenesis were suggested by a series of animal studies [12, 23, 33], the abnormalities in the expression and activation of MerTK on APCs in clinical setting have not been fully elucidated yet. In this study, for the first time, we revealed that the both mMer levels on CD14+ monocyte/macrophages and circulating sMer levels in plasma were significantly elevated in SLE. It is probable that apoptotic cell debris and sustained type I IFN activation in SLE would up-regulate the expression of MerTK to mediate immune-suppressive signaling. Our study clearly showed that the ADAM17 expression in peripheral blood mononuclear cells (PBMC) was positively correlated with sMer levels in plasma (Supplementary Figure 1), which implicated that lower ADAM17 levels might lead to decreased MerTK shedding and sMer production as well as increased mMer level. However, we observed that both sMer and mMer levels increased in SLE patients though ADAM17 expression in PBMCs was significantly lower in SLE patients than in healthy controls. Since the overall MerTK protein expression levels were significantly elevated in SLE patients, mMer levels might increase because of increased total Mer expression and limited shedding by ADAM17, and sMer levels could also increase when total Mer supply significantly increased and ADAM17 shedding was not completely inhibited. Although the protein levels of MerTK increased in SLE, no obvious difference was detected between the MerTK mRNA levels of SLE patients and healthy controls (Figure 1), which suggested that possible variations in posttranscriptional regulation might contribute to different MerTK expression between SLE patients and healthy controls. Future studies on the regulatory mechanisms of MerTK expression would help to reveal the difference.

It was reported that sMer levels were correlated with disease activity of SLE [26, 34]. Consistent with previous studies, our work revealed that sMer levels in plasma were positively correlated to SLEDAI and 24 h proteinuria excretion. SLE patients with severe disease conditions such as higher SLEDAI, elevated 24 h proteinuria excretion, or presence of autoantibodies or lupus nephritis also showed significantly higher plasma sMer levels compared to those without. Similarly, mMer levels on CD14+ monocytes/macrophages were also positively correlated with SLEDAI of SLE patients. In patients with severe disease conditions, mMer levels were significantly increased compared with patients with milder disease. Therefore, the elevation of both mMer and sMer levels could serve as molecular markers of SLE disease activity and indicators of SLE severity. It is likely that the constitutively on-going autoimmune inflammation in SLE is prone to activate MerTK signaling to elicit the negative feedback of immune responses, which induces the overexpression of MerTK and could explain our observation that MerTK expression was more increased in patients with more severe SLE. However, elevated MerTK expression did not effectively inhibit the progression of SLE. Increased plasma sMer might act as a decoy receptor of mMer and inhibited downstream immunosuppressive signaling, and it is necessary to define other defects in the regulation of MerTK signaling in SLE in further investigations.

Recent studies demonstrated that induction of MerTK expression enhanced phagocytosis of apoptotic debris and anti-inflammatory activity of the CD14+CD16+ M2c-like subset of macrophages [28, 35]. In this study, we found that the frequency of circulating CD14+CD16+ monocytes/macrophages in SLE patients significantly decreased compared with healthy controls, while the mMer expression on this subset in SLE patients was significantly up-regulated than in healthy people. The reduced frequency of these anti-inflammatory MerTK expressing CD14+CD16+ monocytes/macrophages might affect the immune homeostasis in SLE. Further studies on the functions of these cells in SLE will be performed in the future.

## 5. Conclusion

In summary, we demonstrated that both the mMer level on circulating CD14+ monocyte/macrophage and sMer level in plasma significantly increased in SLE, and they positively correlated with disease activity and severity. In SLE, the circulating M2c-liked CD14+CD16+ monocyte/macrophage subset, which was reported to play an anti-inflammatory role, showed increased mMer expression but its frequency significantly decreased. The upregulation of MerTK expression may serve as a biomarker of the disease activity and severity of SLE.

## Supplementary Material

ADAM17 has been proved to play a vital role in shedding of MerTK in animal model (Reference 6). To further study the role of ADAM17 in mMer shedding and sMer production, our data demonstrated the correlation between ADAM17 mRNA levels in PBMCs and sMer levels in plasma (supplementary figure 1). In the supplementary figure 2, we have included the representative flow cytometry diagram which showed that higher CD163 expression on CD14+ Mo had higher Mer expression.

## Figures and Tables

**Table 1:** Clinical and laboratory characteristics in patients with SLE and healthy controls.

Clinical characteristics	SLE, *N* (%)^a^	Healthy controls	*P* value^b^
Age	34.63 ± 12.92	35.5 ± 9.75	0.100
Sex (female : male)	95 : 13	34 : 8	0.196
Disease duration (months)	68.16 ± 72.00	NA	
ANA (%)	98/108 (90.74)	NA	
Anti-dsDNA Ab (%)	51/108 (47.22)	NA	
ACL (%)	26/108 (24.07)	NA	
AnuA (%)	55/108 (50.93)	NA	
Sm (%)	26/108 (24.07)	NA	
SSA (%)	45/108 (41.67)	NA	
24 h proteinuria (%)	69/108 (63.89)	NA	
Lupus nephritis (%)	46/108 (42.59)	NA	
Decreased C3 (%)	85/108 (78.70)	NA	
Decreased C4 (%)	68/108 (62.96)	NA	
SLEDAI	9.44 ± 8.32	NA	

SLEDAI: systemic lupus erythematosus disease activity index; ANA: antinuclear antibody; Anti-dsDNA Ab: anti-double strand DNA Antibody; ACL: anticardiolipin antibody; AnuA: antinucleosome antibody; Sm: anti-Sm antibody; SSA: anti-SSA antibody; C3: Complement component 3; C4: Complement component 4. ^a^Values are represented as either mean or number: *N* (%). NA: not applicable. Numerical data were presented as mean ± SD and analyzed using the student's *t*-test or Pearson's Chi-squared test. ^b^ *P* < 0.05 as significant.

**Table 2:** Correlations of the mMer on circulating CD14+ monocytes/macrophages and sMer in plasma with the studied parameters in patients with SLE.

Clinical manifestations	mMer (MFI)		sMer (pg/mL)	
	Spearman's *r*	*P* value	Spearman's *r*	*P* value
SLEDAI	**0.343**	0.026*	**0.229**	0.017*
24 h proteinuria excretion	−0.079	0.621	**0.32**	0.002**
Hb	0.186	0.238	0.077	0.43
Leucocytes	−0.005	0.976	−0.146	0.132
Thrombocytes	−0.023	0.886	0.01	0.917
Anti-dsDNA Ab	0.043	0.786	0.062	0.525
ACL	−0.101	0.53	0.021	0.83
AnuA	0.214	0.174	0.088	0.366
C3	0.097	0.543	−0.171	0.077*
C4	0.178	0.261	−0.051	0.603
IgA	0.214	0.174	−0.002	0.98
IgG	0.066	0.679	−0.052	0.593
IgM	−0.057	0.722	−0.072	0.463
CRP	0.101	0.523	0.06	0.539

SLE: systemic lupus erythematosus; SLEDAI: systemic lupus erythematosus disease activity index; mMer: membrane Mer tyrosine Kinase; sMer: soluble Mer tyrosine kinase; MFI: mean fluorescence intensity; ANA: antinuclear antibody; Hb: haemoglobin; Anti-dsDNA Ab: Anti-double strand DNA Antibody; ACL: anticardiolipin antibody; AnuA: antinucleosome antibody; C3: Complement component 3; C4: Complement component 4; CRP: C-reactive protein. *P < 0.05, **P < 0.01. Spearman's correlation coefficient (*r*) was applied to detect correlation between two types of numerical data.

**Table 3:** mMer and sMer levels in the presence or absence of manifestations in SLE with the studied parameters.

Manifestations	mMer (MFI)			sMer (pg/mL)		
	Presence (*n*)	Absence (*n*)	*P*	Presence (*n*)	Absence (*n*)	*P*
SLEDAI	20.19 ± 3.11	4.42 ± 0.64	0.0001***	2412.8 ± 221.34	1757.9 ± 222.23	0.019*
	(**SLEDAI ≥ 8, ** *n* = 16)	(**SLEDAI < 8, ** *n* = 26)		(**SLEDAI ≥ 8, ** *n* = 59)	(**SLEDAI < 8, ** *n* = 49)	
proteinuria	26.72 ± 3.34	28.02 ± 5.65	0.835	2582.3 ± 225.12	1441.5 ± 139.49	<0.0001***
	(*n* = 28)	(*n* = 14)		(*n* = 69)	(*n* = 39)	
Leukocytopenia	36.14 ± 6.66	22.66 ± 2.44	0.025*	2412 ± 293.28	2044.4 ± 190.94	0.28
	(*n* = 14)	(*n* = 28)		(*n* = 37)	(*n* = 71)	
Thrombocytopenia	34.01 ± 7.81	25.54 ± 3.04	0.253	1699.5 ± 268.83	2304.8 ± 190.2397	0.118
	(*n* = 8)	(*n* = 34)		(*n* = 24)	(*n* = 84)	
Anti-dsDNA Ab	29.16 ± 4.58	26.17 ± 3.70	0.634	2238.4 ± 246.8	2109.4 ± 211.87	0.691
	(*n* = 14)	(*n* = 28)		(*n* = 51)	(*n* = 57)	
ACL	20.61 ± 5.67	28.24 ± 3.21	0.36	2124.6 ± 313.33	2184.8 ± 188.17	0.874
	(*n* = 6)	(*n* = 36)		(*n* = 26)	(*n* = 82)	
AnuA	28.36 ± 4.38	26.33 ± 3.88	0.734	2254.4 ± 243.24	2083.1 ± 210.87	0.597
	(*n* = 17)	(*n* = 25)		(*n* = 55)	(*n* = 53)	
**SSA**	37.06 ± 4.17	16.25 ± 2.13	0.0001***	3196 ± 267.75	1437.7 ± 139.45	<0.0001***
	(*n* = 22)	(*n* = 20)		(*n* = 45)	(*n* = 63)	
**Sm**	36.96 ± 4.84	21.12 ± 3.08	0.006**	3123.6 ± 284.85	1868.1 ± 180.00	0.001***
	(*n* = 16)	(*n* = 26)		(*n* = 26)	(*n* = 82)	
Decreased C3	26.91 ± 3.2	27.94 ± 6.70	0.881	2246.8 ± 184.53	1887.7 ± 325.49	0.363
	(*n* = 32)	(*n* = 10)		(*n* = 85)	(*n* = 23)	
Decreased C4	24.21 ± 3.1	32.46 ± 5.72	0.218	2345.4 ± 226.36	1887.7 ± 325.49	0.117
	(*n* = 27)	(*n* = 15)		(*n* = 68)	(*n* = 40)	
Increased CRP	27.65 ± 4.08	27 ± 3.59	0.925	2387.1 ± 352.56	2083 ± 176.07	0.395
	(*n* = 10)	(*n* = 32)		(*n* = 31)	(*n* = 55)	
Lupus nephritis	33.29 ± 4.07	18.12 ± 2.72	0.004**	2737.1 ± 248.21	1749.8 ± 196.34	0.002**
	(*n* = 25)	(*n* = 17)		(*n* = 46)	(*n* = 62)	

SLE: systemic lupus erythematosus; SLEDAI: systemic lupus erythematosus disease activity index; mMer: membrane Mer tyrosine kinase; sMerTK: soluble Mer tyrosine kinase; MFI: mean fluorescence intensity; Anti-dsDNA Ab: anti-double strand DNA antibody; ACL: anticardiolipin antibody; AnuA: antinucleosome antibody; SSA: anti-SSA antibody; Sm: anti-Sm antibody; C3: Complement component 3; C4: Complement component 4. CRP: C-reactive protein. An independent Student's *t*-test was used for statistical comparison of mMer and sMer levels between the presence and absence of manifestations group in SLE. *P < 0.05, **P < 0.01, and ***P < 0.001.
